# Mussel‐Inspired Adhesive and Tough Hydrogel Based on Silk‐Triggered Dopamine Polymerization for Wound Healing

**DOI:** 10.1002/smmd.70016

**Published:** 2025-08-12

**Authors:** Yu‐Ge Wang, Ting‐Ting Zeng, Hao Wu, Ting‐Ting Zhu, Hui‐Jie Shang, Bo‐Wen Shao, Chun‐Yan Du, Jian‐Jun Yang, Pan‐Miao Liu

**Affiliations:** ^1^ Department of Anesthesiology Pain and Perioperative Medicine The First Affiliated Hospital of Zhengzhou University Zhengzhou China; ^2^ Chiba University Center for Forensic Mental Health Chiba Japan; ^3^ Department of Anesthesiology New Jersey Medical School, Rutgers The State University of New Jersey Newark New Jersey USA; ^4^ Laboratory Animal Center of Zhengzhou University Zhengzhou China

**Keywords:** adhesive hydrogel, polydopamine, Schiff base reaction, silk, wound healing

## Abstract

Tissue engineering is a great alternative to repair and regenerate damaged tissues and organs. Hydrogels are promising materials for tissue repair, but optimizing their various functions—such as adhesion, mechanical properties, and vascularization—to suit the complexity of different organs and tissues remains a significant challenge. In this study, we explore a tough and adhesive polydopamine (PDA)‐silk‐polyacrylamide (PAM) hydrogel inspired by the mussel‐inspired adhesion of PDA and the vascularization potential of silk. Through a Schiff base reaction, self‐polymerization occurs between the free dopamine and the conjugated dopamine on the silk chains, resulting in the formation of a PDA/silk prepolymer. The presence of PDA in the prepolymer endows the resulting PDA‐silk‐PAM hydrogel with excellent adhesiveness, strong mechanical properties, and good water absorption. By adjusting the degree of crosslinking, the hydrogel also demonstrates impressive deformability, making it suitable for engineering thicker and more complex tissues and organs. Moreover, benefiting from the vascularization capabilities of silk and the adhesive properties of PDA, the PDA‐silk‐PAM hydrogel effectively promotes vascularization and accelerates wound healing in full‐thickness skin wounds on the backs of Sprague‐Dawley rats. Overall, our study provides a straightforward approach to create versatile medical hydrogel with strong potential for clinical applications.

## Key Points

- Adhesive PDA‐silk‐PAM hydrogel is inspired by the mussel‐inspired adhesion of PDA and the vascularization potential of silk.
- The Schiff base reaction induces the conjugation of dopamine onto the silk chains, forming a PDA/silk prepolymer.
- The PDA/silk prepolymer endows the hydrogel with excellent adhesiveness, strong mechanical properties, and good water absorption.
- The PDA‐silk‐PAM hydrogel effectively promotes vascularization and accelerates wound healing in full‐thickness skin wounds on the backs of SD rats.

## Graphical abstract

*Adhesive PDA‐silk‐PAM hydrogel* is inspired by the mussel‐inspired adhesion of PDA and the vascularization potential of silk. Such hydrogel exhibits excellent adhesiveness, strong mechanical properties, and good water absorption, and effectively promotes vascularization and accelerates wound healing in full‐thickness skin wounds on the backs of SD rats, providing a straightforward approach to create versatile medical hydrogel with strong potential for clinical applications.

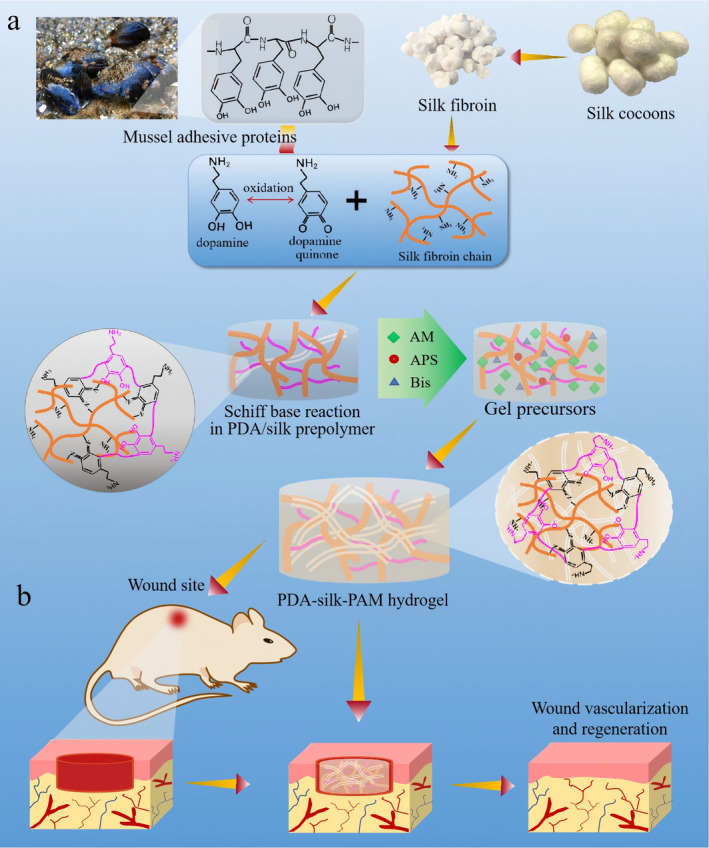

## 1 Introduction

Tissue engineering is a great alternative to repair and regenerate damaged tissues and organs, such as skin, cartilage, and spinal cord [1, 2, 3]. Scaffolds/matrices play a key role in tissue engineering and regenerative medicine. Excellent scaffold/matrix should possess biocompatibility, suitable structure and stiffness, and high surface area, which mimic the structure and biological functions of native extracellular matrix (ECM), for providing a micro‐environment to promote the exchange of biomolecules while not affecting their biological activity. Although there are numerous natural or synthetic components being designed to obtain such scaffolds, there is no universal biomaterial that meets scaffold requirements for all types of tissues [4, 5, 6, 7]. For instance, the current ECM imitating biomaterials achieve more success in the thin constructs such as skin and cartilage, but tend to fail when used to engineer thicker complex organs and tissues that require functional vasculature [8, 9, 10]. Thus, considering vascularization in the design of biological scaffolds is the key to many successful tissue engineering.

Hydrogels with a high percentage of water in their three‐dimensional network have been shown to mimic the native ECM better [11, 12, 13]. Among various types of hydrogels, adhesive hydrogels might be a great alternative for the ECM imitating biomaterial due to their excellent biocompatibility as well as broad adhesion scope, which can be readily adhered to target tissues [14, 15, 16, 17]. Moreover, deformability for adhesive hydrogels might also be an ideal function for ECM imitating biomaterials because deformability allows hydrogels to adhere to many rough or complex tissue surfaces with large deformable areas. In this context, previously reported adhesive hydrogels have achieved partial success in adhesion or deformability, but the simultaneous implementation of all these functions remains a significant challenge. More importantly, previous adhesive hydrogels have primarily focused on repairing tissue's surface or superficial layer, with little attention paid to the regeneration of functional vasculature for complex organs and tissues [15, 18]. Thus, designing ideal adhesive hydrogels with vascularization capacity remains a crucial challenge for the success of many tissue engineering strategies.

Recently, mussel‐inspired adhesive hydrogels with many outstanding advantages have been designed and synthesized for various biomedical applications or tissue engineering. Mussels strongly adhere to virtually all surfaces through their catechol‐enriched byssuses, which are believed to contribute to their adhesion. Owing to the similar catechol groups found in mussel byssus, polydopamine (PDA) has been confirmed to exhibit the same strong adhesion on virtually all substrates, regardless of the surface roughness [19]. Mussel inspired hydrogels are usually synthesized by introducing catechol‐containing proteins or catechol groups into natural or synthetic polymers. The adhesive properties of these hydrogels could only be generated by oxidizing catechol groups using oxidant reagents in alkaline conditions. For example, PDA is formed by oxidizing dopamine with FeCl_3_, NaOI_4_, or O_2_ in alkaline conditions. Note that oxidative reactions often occur rapidly, resulting in short‐term adhesiveness of the obtained hydrogels and single‐use consumption of oxidative agents [17, 20]. In particular, previously reported mussel‐inspired adhesive hydrogels generally endure weak mechanical strength and poor deformability, which impede their ability to meet the toughness and roughness requirements for tissue engineering [21, 22]. Therefore, seeking an ideal oxidation approach for catechol groups to construct adhesive hydrogels with both excellent mechanical properties and deformability remains a significant challenge.

Silk fibroin, a Food and Drug Administration (FDA) approved natural biopolymer material, has been widely used in some biomedical applications and tissue engineering for repairing or replacing damaged or non‐functional tissues and organs. This is due to its many advantages such as good biocompatibility and biodegradability, minimal immune response, good cell adhesion growth, thermal stability and excellent mechanical properties [23, 24, 25]. Silk scaffolds featuring suitable mechanical properties and a nanofibrous structure have been demonstrated to induce endothelial cell differentiation and neovascularization through physical stimuli alone without requiring growth factors [26]. Silk‐based biomaterials were also shown good fibroblast proliferation, improved neovascularization and faster and better tissue repair and regeneration in a rat model [27, 28]. Therefore, incorporating silk into ideal mussel‐inspired adhesive hydrogels is a very promising approach for fabricating ECM imitating biomaterials with vascularization capacity.

**FIGURE 1:**
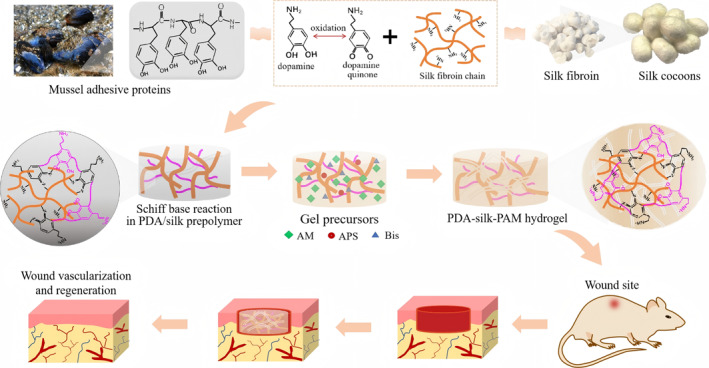
Design strategy for the preparation of PDA‐silk‐PAM hydrogel and the application of the PDA‐silk‐PAM hydrogel on wound healing.

In this study, we propose a new class of polydopamine‐silk fibroin‐polyacrylamide (PDA‐silk‐PAM) hydrogel that exhibits excellent adhesiveness, strong mechanical properties, facile deformability and performing vascularization capacity, which is based on the mussel‐inspired adhesion mechanism and the vascularization capacity of silk. As shown in Figure 1, the hydrogel was produced through a two‐step procedure. First, dopamine (DA) was intercalated into the chains of silk, with the amino groups of silk covalently linked to the ketone groups of dopamine quinone via a Schiff base reaction. Then, a self‐polymerization occurred between the free dopamine and the conjugated dopamine on the silk chains, resulting in a PDA/silk prepolymer with free catechol groups. Second, acrylamide (AM) monomers were added and polymerized in situ by free radical polymerization in the presence of initiator and cross‐linker, forming a free‐standing and adhesive PDA‐silk‐PAM hydrogel. The excellent adhesiveness and deformability of the PDA‐silk‐PAM hydrogel could be flexibly controlled by adjusting the amount of cross‐linker. The double crosslinked structure endows the hydrogels with a loose porous structure, allowing a higher percentage of water in its three‐dimensional network. Due to the mussel‐inspired adhesion of PDA, the hydrogel exhibits repeatable and self‐healing adhesiveness capacity and could adhere to various surfaces. Meanwhile, cytotoxicity and hemolysis experiments revealed that the PDA‐silk‐PAM hydrogel possessed excellent biocompatibility and could accelerate wound healing without hemolysis, redness or other adverse reactions. Furthermore, histological analysis of the healed wounds demonstrated that the PDA‐silk‐PAM hydrogel has significant vascularization capacity, which might be the crucial reason for the repair and regeneration of skin tissue.

## 2 Results and Discussion

**FIGURE 2:**
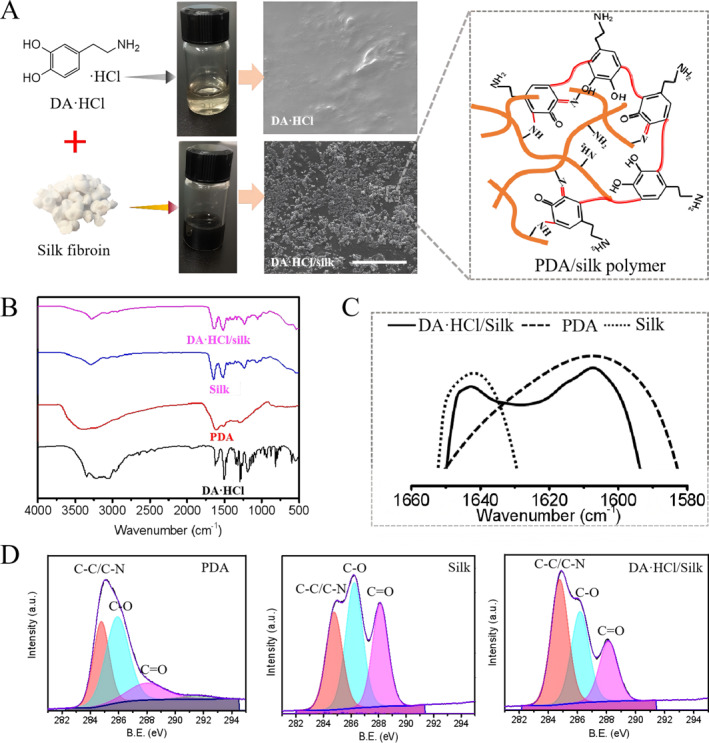
Preparation and characterization of PDA/Silk polymer. (A) Schematic and mechanism of preparing PDA/Silk polymer. (B) FT‐IR spectra of DA·HCl, PDA, silk and DA·HCl/Silk. (C) FT‐IR spectra with 1580–1660 cm^−1^ of PDA, Silk and DA·HCl/silk. (D) XPS high‐resolution spectra: C1s peak of PDA, silk, and DA·HCl/Silk, respectively.

Prior to the hydrogels, dopamine was first grafted onto silk fibroin chains to form a Schiff base, which promotes the self‐polymerization between free and conjugated dopamine on silk chains, resulting in a PDA/silk prepolymer [27]. During this process, silk fibroin not only acted as a gelatin backbone to generate a double crosslinked structure but also served as the initiator for PDA formation. As shown in Figure 2A, when dopamine hydrochloride (DA·HCl) was mixed with a silk fibroin‐containing solution for 18 h, the solution turned inky black, indicating the presence of PDA in this system. This did not occur when DA·HCl was mixed with the silk fibroin‐free water. Characterization of FESEM (Field emission scanning electron microscopy) further demonstrated that the inky black color of the DA·HCl/silk solution contained many agglomerates, which might originate from the generation of PDA. To confirm this, FT‐IR measurements in Figure 2B showed that the DA·HCl/silk appeared an indole‐related structure located around 1600 cm^−1^, a characteristic peak of PDA that is nonexistent in DA HCl. Besides, the absorption band between 800 cm^−1^ and 700 cm^−1^, attributed to the aromatic hydrogen and aromatic nucleus, was weaker in DA·HCl/silk than in DA·HCl, which may be due to the oxidation reaction of the aromatic ring of DA under the catalysis of silk fibroin. Moreover, DA·HCl/silk had the same characteristic peaks of PDA and silk, including the vibration of aromatic ring from PDA at 1610 cm^−1^ and the amido group from silk at 1512 cm^−1^ (Figure 2C). X‐ray photoelectron spectroscopy (XPS) results in Supporting Information S1: Figures S1 and 2D presented the C1s peaks, which were divided into three peaks at 284.6, 286.0, and 287.63 eV, corresponding to C–N/C–C, C–O, and C=O, respectively. These results indicated that the C–C or C–N bonds increased in the DA·HCl/silk solution, and silk promoted the oxidative autopolymerization of DA·HCl, forming more covalent bonds during the process. This may due to the alkaline amino acid lysine in silk, which could induce a Michael addition reaction. Overall, these results indicate that DA·HCl/silk system forms a PDA/silk prepolymer, which is induced by the formation of a PDA/silk Schiff base.

**FIGURE 3:**
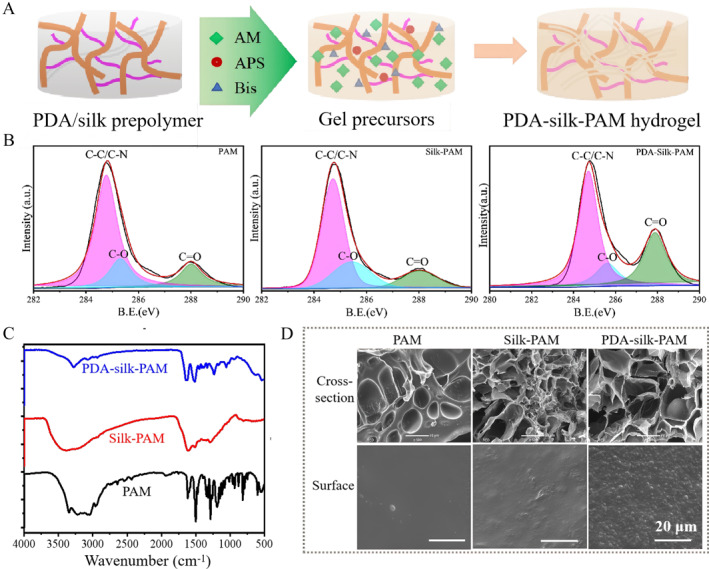
Preparation and characterization of PDA‐silk‐PAM hydrogel. (A) Schematic and mechanism of preparing PDA‐silk‐PAM hydrogel. (B) XPS high‐resolution spectra: C1s peak of PDA, silk, and DA·HCl/Silk, respectively. (C) FT‐IR spectra of PDA‐silk‐PAM, Silk‐PAM and PAM hydrogels. (D) SEM images of PDA‐silk‐PAM, Silk‐PAM and PAM hydrogels on the surface and cross‐section. Scale bar: 20 μm.

Subsequently, acrylamide and the crosslinking agent were added into the PDA/silk prepolymer reaction system, and APS was directly used as the initiator for the synthesis of PDA‐silk‐PAM hydrogels. After a reaction in a 60°C chamber for 3 h, the liquid gel precursor solidified into a hydrogel (Figure 3A). The elemental composition, including C1s, O1s, and N1s, could be observed via XPS survey spectra (Supporting Information S1: Figure S2). The C1s peaks in the XPS spectrum were divided into three peaks at 284.6, 286.0, and 287.63 eV, which were attributed to C–N/C–C, C‐sO, and C=O, respectively (Figure 3B). The increase in C=O bonding in PDA‐silk‐PAM hydrogels indicated that PDA doping increased more quinone groups due to the oxidation of phenol hydroxyl group, which may play a crucial role in the adhesion function. FT‐IR measurements further characterized the PDA‐silk‐PAM, silk‐PAM and PAM hydrogels, respectively. Compared with silk‐PAM and PAM hydrogels, PDA‐silk‐PAM hydrogels appeared an obvious PDA characteristic peak of the indole‐related structure at around 1600 cm^−1^ (Figure 3C). Thus, PDA‐silk‐PAM hydrogels appeared a transparent brown color. Observed by SEM (Scanning Electron Microscopy) (Figure 3D), the cross‐section of the PDA‐silk‐PAM hydrogel showed more uniform and larger holes (∼50 μm) in its three‐dimensional structure compared to the Silk‐PAM and PAM hydrogels. This porous structure improved the mechanical properties, potential for enhanced bioactivity, and structural stability in the PDA‐silk‐PAM hydrogel. Additionally, there were many small particles inserted on the surface of the PDA‐silk‐PAM hydrogel, which might be the PDA granules. These results indicated that the PDA‐silk‐PAM hydrogel was obtained successfully through the thermal co‐polymerization of PDA/silk prepolymer and acrylamide.

**FIGURE 4:**
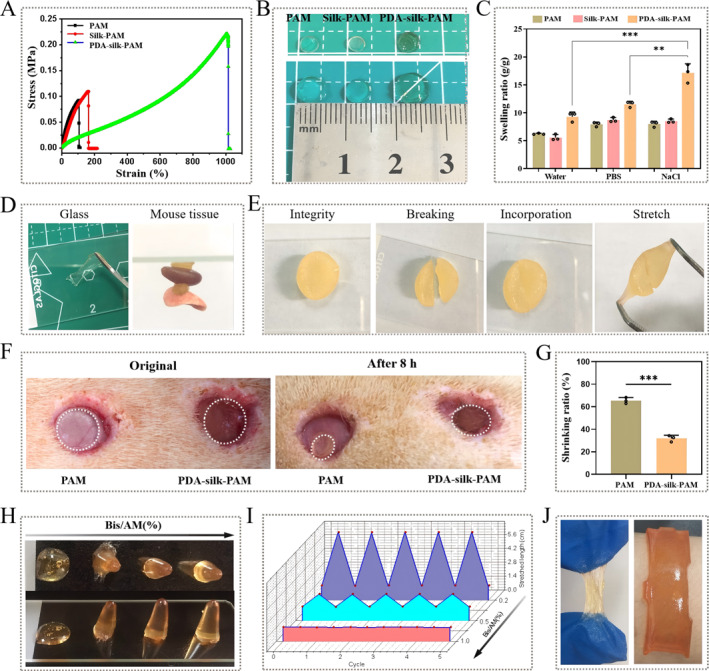
Mechanical, hydroscopicity and adhesion properties of PDA‐silk‐PAM hydrogel. (A) Typical tensile stress–strain curves of PAM, Silk‐PAM and PDA‐silk‐PAM hydrogels. (B) Digital photos of PAM hydrogel, Silk‐PAM hydrogel and PDA‐silk‐PAM hydrogels before and after being fully immersed in water. (C) Swelling ratio of PAM, Silk‐PAM and PDA‐silk‐PAM hydrogels fully immersed in water, PBS and NaCl solution. (Statistical significance was tested using a one‐way ANOVA with Bonferroni's multiple comparisons test; *n* = 3 for each group; the data represented means ± SEM; *p < 0.05; **p < 0.01; ***p < 0.001). (D) Adhesion of PDA‐silk‐PAM hydrogels on glass and mouse tissue. (E) Self‐healing process composed of two halves of block from PDA‐silk‐PAM hydrogel. Digital photos (F) and statistical bar chart (G) of PDA‐silk‐PAM hydrogel and PAM hydrogel after 8 h adhere in a skin wound of rat. (Statistical significance was tested using an unpaired *t* test; *n* = 3 for each group; the data represented means ± SEM; *p < 0.05; **p < 0.01; ***p < 0.001). (H) Digital photos of PDA‐silk‐PAM hydrogels with the crosslinking degrees (Bis/AM) of 0.1%, 0.2%, 0.5% and 1.0% from left to right. (I) The stretch length adhering glass of the PDA‐silk‐PAM hydrogels with the crosslinking degrees (Bis/AM) of 0.2%, 0.5% and 1.0%. (J) The digital photos of PDA‐silk‐PAM hydrogels with the crosslinking degrees (Bis/AM) of 0.1% (left) and 1.0% (right).

As shown in Supporting Information S1: Figure S3, the PDA‐silk‐PAM hydrogel could be stretched to at least 5 times its initial length and recovered to its initial length integrally in 1 min. Figure 4A shows the typical tensile stress‐strain curves of the hydrogels in uniaxial tensile tests at a crosshead speed of 120 mm min^−1^. The PDA‐silk‐PAM hydrogel displayed a maximum tensile strain of 1000%, in sharp contrast to the 114% maximum strain of the PAM hydrogel and 164% maximum strain of the Silk‐PAM hydrogel. Meanwhile, the tensile strength of the PDA‐silk‐PAM hydrogel was about 220 kPa, much higher than that of PAM hydrogel (92 kPa) and Silk‐PAM hydrogel (110 kPa). These results indicated that the mechanical performance of the PDA‐silk‐PAM hydrogels synergistically depended on the DA and silk contents. Water content is also a critical factor for hydrogels used in tissue engineering. Figure 4B showed that PAM hydrogel, Silk‐PAM hydrogel and PDA‐silk‐PAM hydrogel swelled to different degrees after being fully immersed in water. The swelling ratio was further calculated by weighing the mass of hydrogel before and after swelling. Figure 4C showed that PDA‐silk‐PAM hydrogel had the highest swelling ratio than that of Silk‐PAM hydrogel and PAM hydrogel in water, PBS and NaCl solution. Especially in normal saline‐NaCl solution, the PDA‐silk‐PAM hydrogel displayed a swelling ratio of 17.11, much higher than that of the PAM hydrogel (7.98) and the Silk‐PAM hydrogel (8.53). Meanwhile, the PDA‐silk‐PAM hydrogel in normal saline‐NaCl solution also presented the highest swelling ratio compared to its swelling ratios in water (9.31) and PBS solution (11.54). Due to its mussel‐inspired adhesion to a wide range of surfaces, PDA‐silk‐PAM hydrogel also exhibited good adhesion to both hydrophobic and hydrophilic surfaces. The hydrogel exhibited strong adhesion to natural surfaces such as glass, leaves, paper, plastomer, aluminum, and fresh organ tissues with tissue fluid (Supporting Information S1: Figures S4 and 4D). Moreover, the self‐healing capability of hydrogel is an important property for its application in wound dressings. To affirm this, we cut the integral cylindrical PDA‐silk‐PAM hydrogel into two pieces and then patched them into a whole one. The two halves of the hydrogel that were physically brought into contact could recombine into a new piece, with heterogeneous colors observed under the optical microscopy. Meanwhile, this hydrogel could withstand stretching by tweezers without cracks, indicating its superior self‐healing property (Figure 4E). Besides, benefiting from its excellent water absorbing capacity and adhesion property, PDA‐silk‐PAM hydrogel exhibited a lower shrinking percentage (32.2%) than PAM hydrogel (65.4%) after 8 h of application to a skin wound of rat (Figure 4F,G). These results demonstrate that the PDA/silk prepolymer endows the PDA‐silk‐PAM hydrogel with superior properties, including mechanical properties, water content and adhesion.

The morphology deformation regulation capacity allows adhesive hydrogels to meet a wide variety of requirements, such as tissue adhesives, wound dressings, and surgical sealants [29, 30, 31]. In order to obtain adhesive PDA‐silk‐PAM hydrogels with different flowability, four kinds of hydrogels were obtained by adjusting the crosslinking degrees (Bis/AM) to 0.1%, 0.2%, 0.5%, and 1.0% during the synthesis process (Supporting Information S1: Figure S5). After thermal polymerization at 60°C for 3 h, the 0.1% cross‐linked PDA‐silk‐PAM hydrogels presented a flowing liquid state, while the 0.2%, 0.5%, and 1% PDA‐silk‐PAM hydrogels were in stable solid states (Figure 4H). The adhesion and stretch ability of these solid PDA‐silk‐PAM hydrogels with a height of 1.4 cm were tested using a slide press‐pull procedure. The length of the 0.2% cross‐linked PDA‐silk‐PAM hydrogel after sticking on the slide was significantly higher (6.9 cm) than that of the 0.5% (2.7 cm) and 1.0% (1.4 cm) hydrogels (Figure 4I). The change in flowability as a function of crosslinking degree was further observed in the cross‐sectional images of microscope. The hydrogel films were placed on U‐groove line patterns for 12 h in an ambient environment. The PDA‐silk‐PAM hydrogel films with 0.5% and 1.0% crosslinking degrees did not deform along the patterned surface of the mold due to their low flowability. In contrast, the films with 0.2% and 0.1% crosslinking degrees completely filled the grooves with no gap, demonstrating sufficiently high flowability (Supporting Information S1: Figure S6A). Therefore, the adhesive PDA‐silk‐PAM hydrogels can be made into either a flowing glue or a super adhesive elastomer (Figure 4J). The as‐obtained 0.2% PDA‐silk‐PAM hydrogel showed stretchable and adhesive properties, and could cover a phalangeal joint completely (Supporting Information S1: Figure S6B).

**FIGURE 5:**
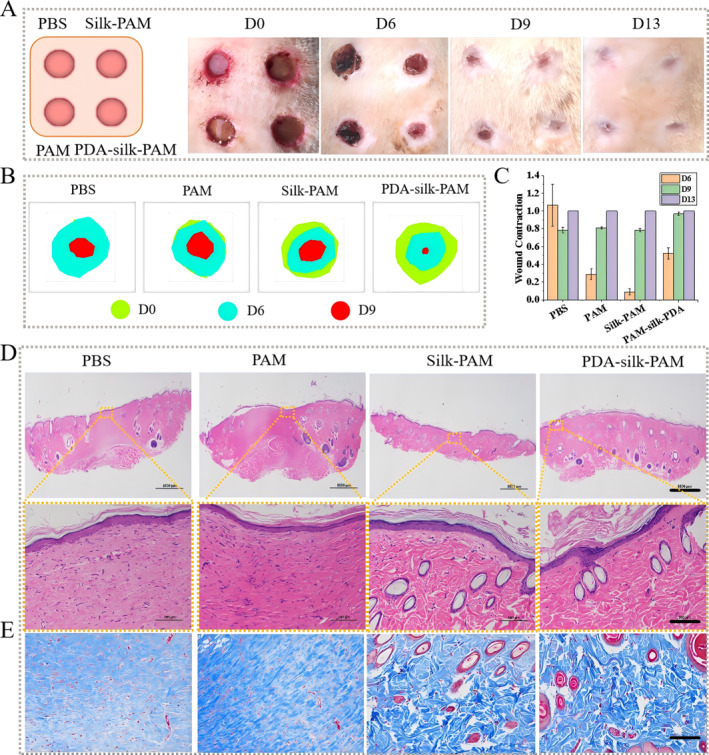
In vivo study using the hydrogels for the treatment of skin wounds on the back of rats. (A) Schematic photo and digital photos of wound healing at 0, 6, 9, and 13 days. (B) Superimposed heat map analysis of wound area at 0, 6, and 9 days. (C) Quantitative analysis of wound healing. (D) Histomorphology evaluation of wound regeneration with the treatment of PBS, PAM hydrogel, Silk‐PAM hydrogel and PDA‐silk‐PAM hydrogel on the 16th day. (E) Masson's staining evaluation of wound regeneration for PBS, PAM hydrogel, Silk‐PAM hydrogel and PDA‐silk‐PAM hydrogel on the 16th day. Data are presented as the mean ± SEM, *n* = 3. (A–E) Data with different symbols that have significant differences. Original figures, scale bar: 1000 μm for panel (upper images of D); 100 μm for panels (lower imges of D and E).

To implement this assay, full‐thickness skin wounds were created on Sprague–Dawley (SD) rats, and hydrogels were directly applied by covering the skin wounds. As shown in Figure 5A, the optical images of the wounds showed that the PDA‐silk‐PAM hydrogel‐treated group showed a faster healing process than the other three groups. The wound healing superimposed heat map also showed the same results (Figure 5B). The wound contraction ratio of the PDA‐silk‐PAM hydrogel group increased from 0.0 to 96.86 ± 1.8% from day 0 to day 9 (Figure 5C), and no scar could be seen in any of the four groups in day 13 (Figure 5C). Importantly, the presence of an intact epidermal layer has been a key indicator for evaluating the reepithelialization in wound repair. Hematoxylin and eosin (H&E) staining was employed to evaluate the regeneration of the epidermis and the production of skin appendages on the 16th day (Supporting Information S1: Figure S7). As shown in Figure 5D, the PDA‐silk‐PAM hydrogel group showed more blood vessels and hair follicles compared to the other three groups. In addition, collagen deposition plays important roles in repairing tissue structure and function during the wound healing process. Masson images (Figure 5E) showed substantial collagen fibers in the PDA‐silk‐PAM hydrogel group, further confirming its excellent wound‐healing efficiency based on its excellent adhesion property, porous structure and high water content effect.

**FIGURE 6:**
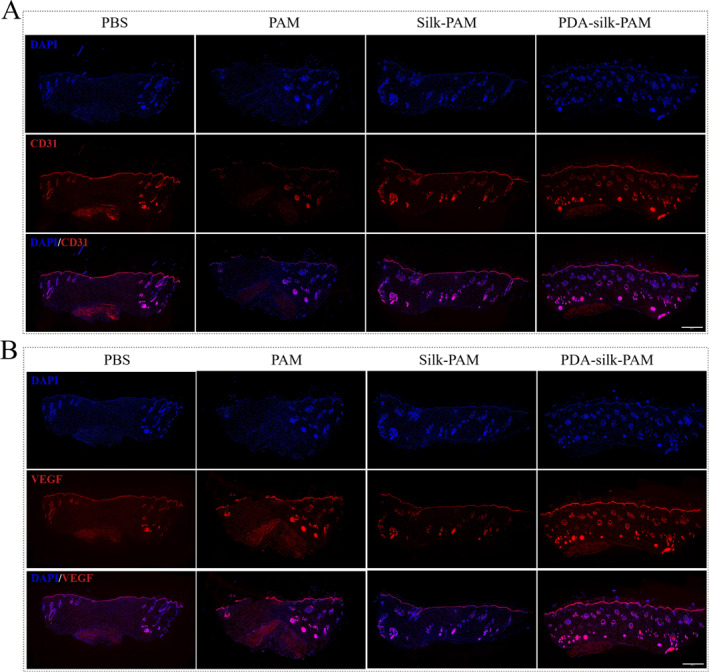
Immunofluorescence staining of CD31 (A) and VEGF (B) of PBS, PAM hydrogel, Silk‐PAM hydrogel and PDA‐silk‐PAM hydrogel at the 16th day on the back of rats. Scale bar: 1000 μm.

Moreover, the vascular endothelial growth factor (VEGF) has an effect on the vascular endothelial cell migration, proliferation and angiogenesis. CD31, a transmembrane protein expressed during early angiogenesis, is mainly used to demonstrate the presence of endothelial cell tissue and can be used to assess angiogenesis [32]. On day 16, the PDA‐silk‐PAM hydrogel as well as Silk‐PAM hydrogel group showed higher expression of VEGF and CD31 compared with the PBS and PAM groups. Specifically, the PDA‐silk‐PAM group had the highest expression of CD31 (Figure 6A) and VEGF (Figure 6B). These immunofluorescence staining results indicated that the silk based PDA‐silk‐PAM and Silk‐PAM hydrogel can up‐regulate the expression of VEGF and CD31, thereby promoting vascularization and wound healing.

Prior to the usage of PDA‐silk‐PAM hydrogels in vivo for wound healing, it was important to investigate their biocompatibility. A cell counting kit (CCK8) assay was performed to detect the viability of MRC5 and RS1 cells in contact with PDA‐silk‐PAM hydrogels. As shown in Supporting Information S1: Figure S8A,B, MRC5 and RS1 cells both showed good viability for 1, 3, 5, and 7 days of incubation with control, PAM hydrogels, Silk‐PAM hydrogels and PDA‐silk‐PAM hydrogels. Notably, the cellular safety of PDA‐silk‐PAM hydrogels was independent of co‐culture time. In addition, hemolytic assay was considered to estimate the blood compatibility of PDA‐silk‐PAM hydrogels. All the hydrogels showed no obvious hemolysis after 4 h of co‐incubation (Supporting Information S1: Figure S8C,D). Furthermore, to fully understand the biocompatibility in vivo, immunological analyses of inflammatory factors of IL‐6, IL‐1β, and TNF‐α were assessed. The skin wounds treated with the PDA‐silk‐PAM hydrogels showed the same or even lower expression in IL‐6, IL‐1β, and TNF‐α comparing with those treated with PBS, confirming their good biocompatibility and potential in wound‐healing (Supporting Information S1: Figure S9). As previously mentioned, PDA‐silk‐PAM hydrogels exhibited good biocompatibility both in vivo and in vitro.

## 3 Conclusion

In summary, we present a tough and adhesive PDA‐silk‐PAM hydrogel inspired by the mussel‐inspired adhesion of PDA and the vascularization potential of silk. Through a Schiff base reaction, self‐polymerization occurs between free dopamine and conjugated dopamine on silk chains, yielding a PDA/silk prepolymer. This prepolymer imparts the hydrogel with excellent adhesiveness, robust mechanical properties, and good water absorption. By adjusting the crosslinking degree, the hydrogel also demonstrated enhanced deformability, enabling the engineering of thicker and more complex tissues. Leveraging the vascularization capacity of silk and the adhesive properties of PDA, the PDA‐silk‐PAM hydrogel effectively promotes vascularization and accelerates wound healing in full‐thickness skin wounds in SD rats. Overall, our study introduces an efficient strategy for developing a versatile medical hydrogel with strong potential for clinical applications.

The PDA‐silk‐PAM hydrogel exhibited excellent mechanical properties, adhesiveness, and biocompatibility, making it a promising candidate for various tissue engineering applications, including cartilage, bone, and nerve regeneration. The hydrogel promoted significant vascularization and tissue regeneration as evidenced by the formation of new blood vessels and hair follicles. This suggests that the hydrogel not only supports wound healing but also facilitates long‐term tissue repair and regeneration. More importantly, by varying the crosslinking degree, the mechanical properties and flowability of the hydrogels could be tailored to meet specific clinical needs. Besides, the high water content and porous structure could be utilized for controlled drug release, making it suitable for local drug delivery systems in cancer therapy or chronic wound management. Given the high water absorption and adhesive properties, the hydrogel could be adapted for burn wound dressings to maintain a moist environment and promote rapid healing. The strong adhesion and mechanical strength also allowed the hydrogel be beneficial in surgical procedures, such as hemostasis or tissue repair, where a durable and biocompatible material is required.

However, our study focused on short‐term outcomes (up to 13 days) for wound healing. Long‐term follow‐up studies are needed to assess the durability and long‐term effects of the PDA‐silk‐PAM hydrogel on tissue regeneration and overall health. Although our comprehensive in vitro and in vivo studies demonstrate that the PDA‐silk‐PAM hydrogel is highly biocompatible, more comprehensive evaluation of immune responses, including long‐term immune compatibility and some inflammatory markers, is required. Future studies should include detailed immunological analyses to fully understand the hydrogel's interaction with the immune system with minimal inflammatory or immune responses. The exact mechanisms by which the hydrogel promotes vascularization and wound healing are not fully elucidated. Further studies are needed to explore the molecular and cellular pathways involved in these processes.

## 4 Experimental Section/Methods

### 4.1 Preparation of Hydrogels

The PDA‐silk‐PAM hydrogels were synthesized through the following procedures: (1) silk‐triggered DA oxidation: Silk fibroin was dispersed into a DA solution to form a silk/DA suspension. The suspension was vigorously stirred for 24 h to facilitate the intercalation and oxidation of DA. (2) Hydrogel formation: Acrylamide (AM), ammonium persulfate (APS), and *N*,*N*‐methylenebis (acrylamide) (Bis) were added to the DA/silk suspension at room temperature under stirring. After stirring for 5 min, the suspension was then immersed in a 60°C water bath. After 3 h, the AM was polymerized to form PDA‐silk‐PAM hydrogels.

### 4.2 Microstructure of Hydrogels

The microstructures of the PDA‐silk‐PAM, Silk‐PAM and PAM hydrogels were examined using a SEM (JSM 6390, JEOL, Japan). Before examination, the hydrogels were freeze‐dried. The dried hydrogels were cut to expose their inner structure, and the cross‐section was observed by SEM.

### 4.3 Rheological Experiments

The mechanical properties of the PAM, Silk‐PAM, and PDA‐silk‐PAM hydrogels were tested on MTS Exceed E42 electronic universal testing machine equipped with pneumatic clamps (DQB203B) at room temperature. For tensile test, hydrogels with a thickness of 0.5 mm were cut into rectangle (40 mm in length and 10 mm in width). The rate of extension was fixed at 50 mm min^−1^ for tensile test and loading‐unloading test.

### 4.4 Adhesion Tests

Tensile adhesion testing was performed to measure the adhesive strength of the PDA‐silk‐PAM hydrogels. The hydrogels were applied to the surface of the specimens with a bonding area of 10 mm × 10 mm. The substrates that we chose for investigation were glass, plastomer, Aluminum, leaf, and paper, representing hydrophilic, hydrophobic, and metal materials, etc. Mouse tissue was chosen for mimicking adhesion on human tissue. The adhesion test was immediately conducted once the hydrogel was attached on the substrate surfaces, which did not need curing time.

### 4.5 Mechanical Properties Testing

The tensile and compression tests were performed on a universal test machine (Instron 5567, USA). For tensile testing, the specimens were 41 mm in length and 3 mm thick. Details are provided in the Supporting Information.

### 4.6 XPS Analysis

In order to analyze the structure of DA after oxidizing by different agents, PDA, silk, DA‐HCl, PAM, Silk‐PAM, and PDA‐silk‐PAM were prepared. The chemical compositions were then measured using an X‐ray photoelectron spectrometer (Kratos, Axis Ultra DLD, UK).

### 4.7 FTIR Measurement

The chemical structure was acquired by using Fourier transform infrared spectroscopy (FTIR, Nicolet iS20, Termo).

### 4.8 In Vitro Cell Culture

The PDA‐silk‐PAM, Silk‐PAM and PAM hydrogels were used to culture the cells. MRC5 and RS1 (Stem Cell Bank, Chinese Academy of Sciences, SCSP‐515) were cultured in Dulbecco minimum essential medium (DMEM, HyClone, USA) with 10% fetal bovine serum (HyClone) and 1% penicillin‐streptomycin solution (HyClone) in a CO_2_ incubator at 37°C. Before cell seeding, the hydrogels with a diameter of 7 mm and a thickness of 2.5 mm were first purified in PBS and sterilized with 75% ethanol for 24 h. The hydrogels were then immersed in DMEM and swelled to an equilibrium state. Cells in a growth phase were seeded on the hydrogels with a density of 5 × 10^4^ cells/mL. The cell growth on the hydrogels was evaluated by CCK8 (Sigma, USA) assay.

### 4.9 In Vivo Wound Healing

Full‐thickness skin wounds were created on the dorsal area of rats. The defects were treated according to the types of specimens: (1) PAM, (2) Silk‐PAM, (3) PDA‐silk‐PAM, and (4) PBS as a control group. Four parallel specimens of each type of hydrogels were tested. The surgical procedure followed our previous study.

### 4.10 Immunohistochemistry

Mice were deeply anesthetized with isofurane and slowly perfused with phosphate‐buffered saline (PBS) and 4% paraformaldehyde (PFA). Skins were carefully removed and postfixed in 4% PFA overnight, then dehydrated with 20% and 30% sugar in PBS and coronally cut into 25 μm‐thick brain slices using a cryostat (CM1950, Leica). The sections were blocked with 5% normal goat serum and 0.3% Triton X‐100 in PBS for 1 h at room temperature followed by incubation with primary antibody against CD31 (1 μg/mL, A2104, Abcam) or VEGF (5 μg/mL, A12303, Abcam) overnight at 4°C. Next, wash the sections three times with PBS before incubating them with the goat anti‐rabbit IgG‐Cy_3_ (3.75 μg/mL, 115165045, Jackson ImmunoResearch) or goat antimouse IgG‐FITC (15 μg/mL, GB21303, Servicebio) for 2 h at room temperature. Finally, 10 μg/mL 4′, 6‐diamidino‐2‐phenyl‐indole (DAPI) (G1012, Servicebio) immersed the slices for 5 min for nuclear staining for localization. Fluorescence microscopy images were collected by a confocal scanning microscope (A1 MP+, Nikon).

## Author Contributions

P.‐M.L. conceived the idea. Y.‐G.W. and B.‐W.S. conducted experiments. T.‐T.Z. and T.‐T.Zhu carried out data analysis and wrote the manuscript. T.‐T.Z., H.W., and T.‐T.Zhu revised the manuscript. H.‐J.S., C.‐Y.D., P.‐M.L., and J.‐J.Y. contributed to the scientific discussion of the article.

## Ethics Statement

The Animal Ethics Committee of the Laboratory Animal Center of Zhengzhou University approved all animal experimental procedures (ZZU‐LAC20211112[17]).

## Conflicts of Interest

The authors declare no conflicts of interest.

## Supporting information

Supporting Information S1

## Data Availability

The data that support the findings of this study are available from the corresponding author upon reasonable request.
