# Supplementary material for: Mussel‐Inspired Adhesive and Tough Hydrogel Based on Silk‐Triggered Dopamine Polymerization for Wound Healing
Source: Smart Med. 2025 Aug 12;4(3):e70016. doi: 10.1002/smmd.70016 (PMC12362727; doi:10.1002/smmd.70016)
Supplement: Supplementary file 1 — Supporting Information S1 [file SMMD-4-e70016-s001.docx]

Supporting Information

Mussel-Inspired Adhesive and Tough Hydrogel Based on Silk-Triggered Dopamine Polymerization for Wound Healing

*Yu-Ge Wang, Ting-Ting Zeng, Hao Wu, Ting-Ting Zhu, Hui-Jie Shang, Bo-Wen Shao, Chun-Yan Du^*^, Jian-Jun Yang^*^, Pan-Miao Liu^*^*


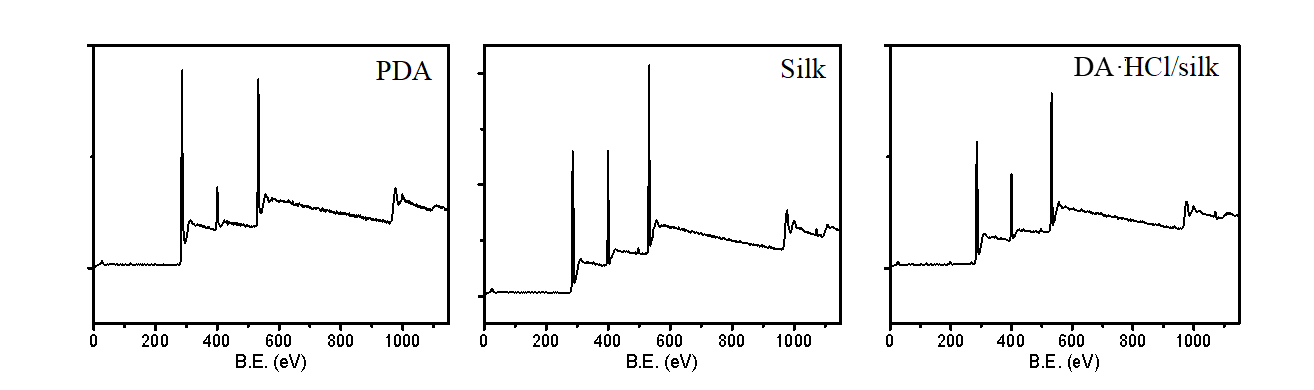


**Figure S1.** XPS spectra of PDA, silk, and DA•HCl/Silk, respectively.


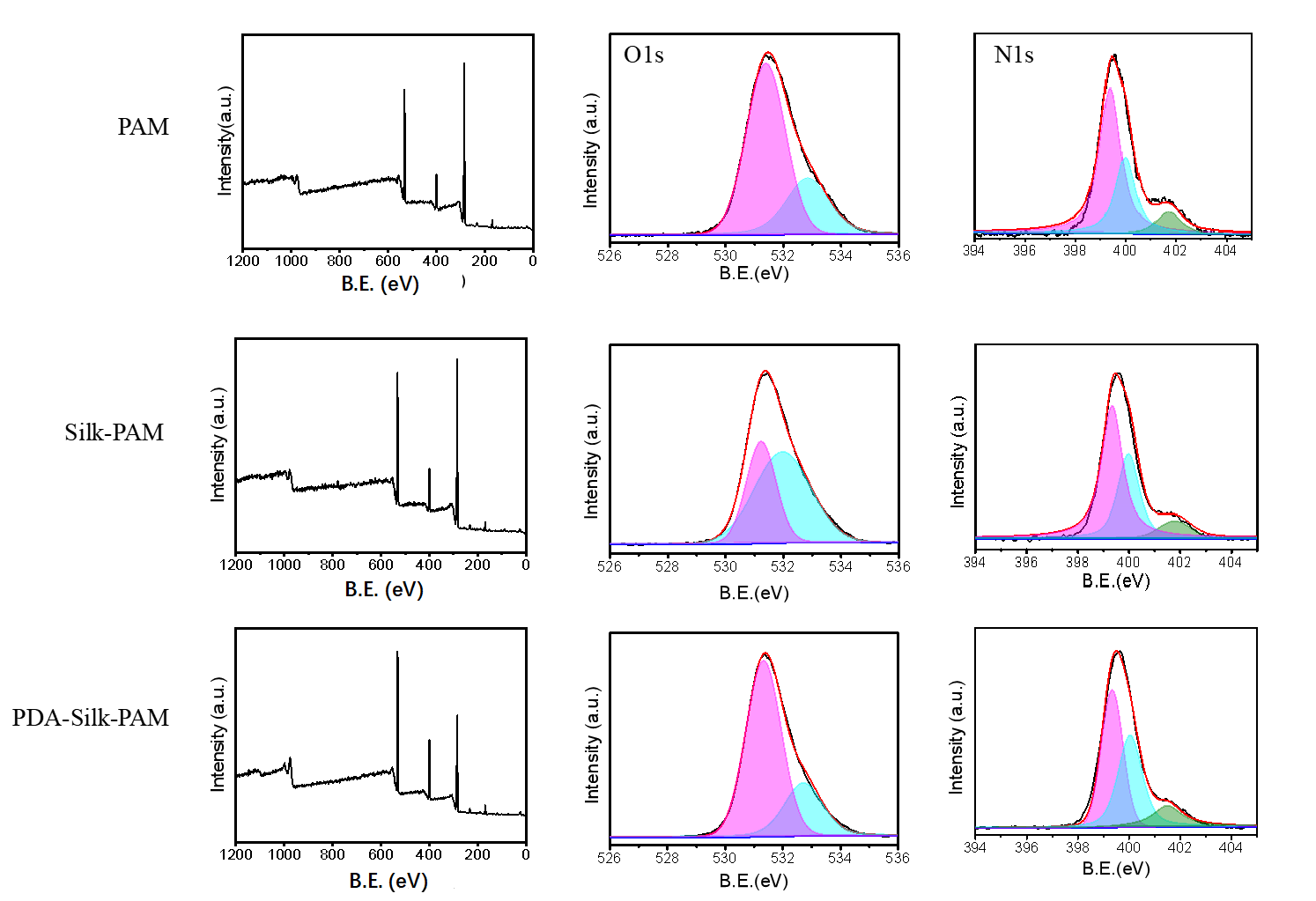


**Figure S2.** XPS original and high-resolution spectra: O1s and N1s peak of PAM, Silk-PAM, and PDA-Silk-PAM, respectively.


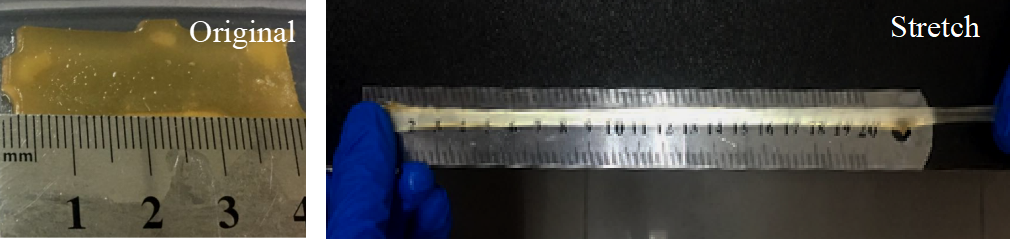


**Figure S3.** Digital photos of the tensile test of PDA-silk-PAM hydrogel, showing its high stretchability.


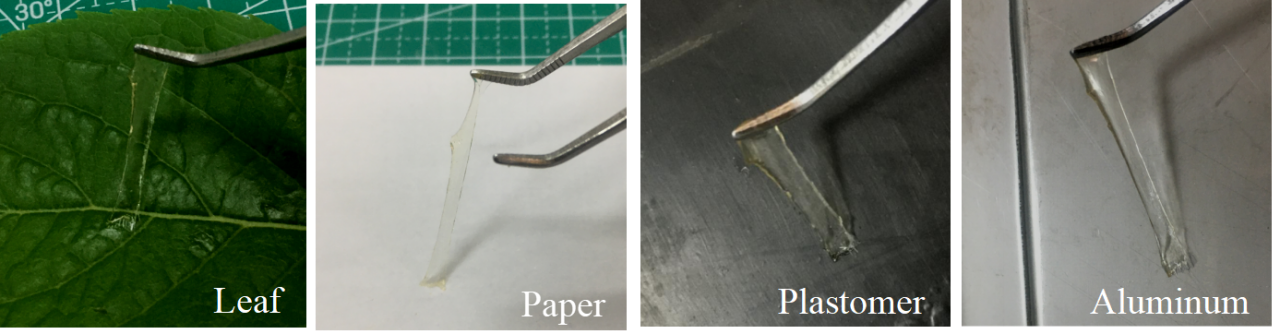


**Figure S4.** Adhesion of PDA-silk-PAM hydrogels on leaf, paper, plastomer and aluminum.


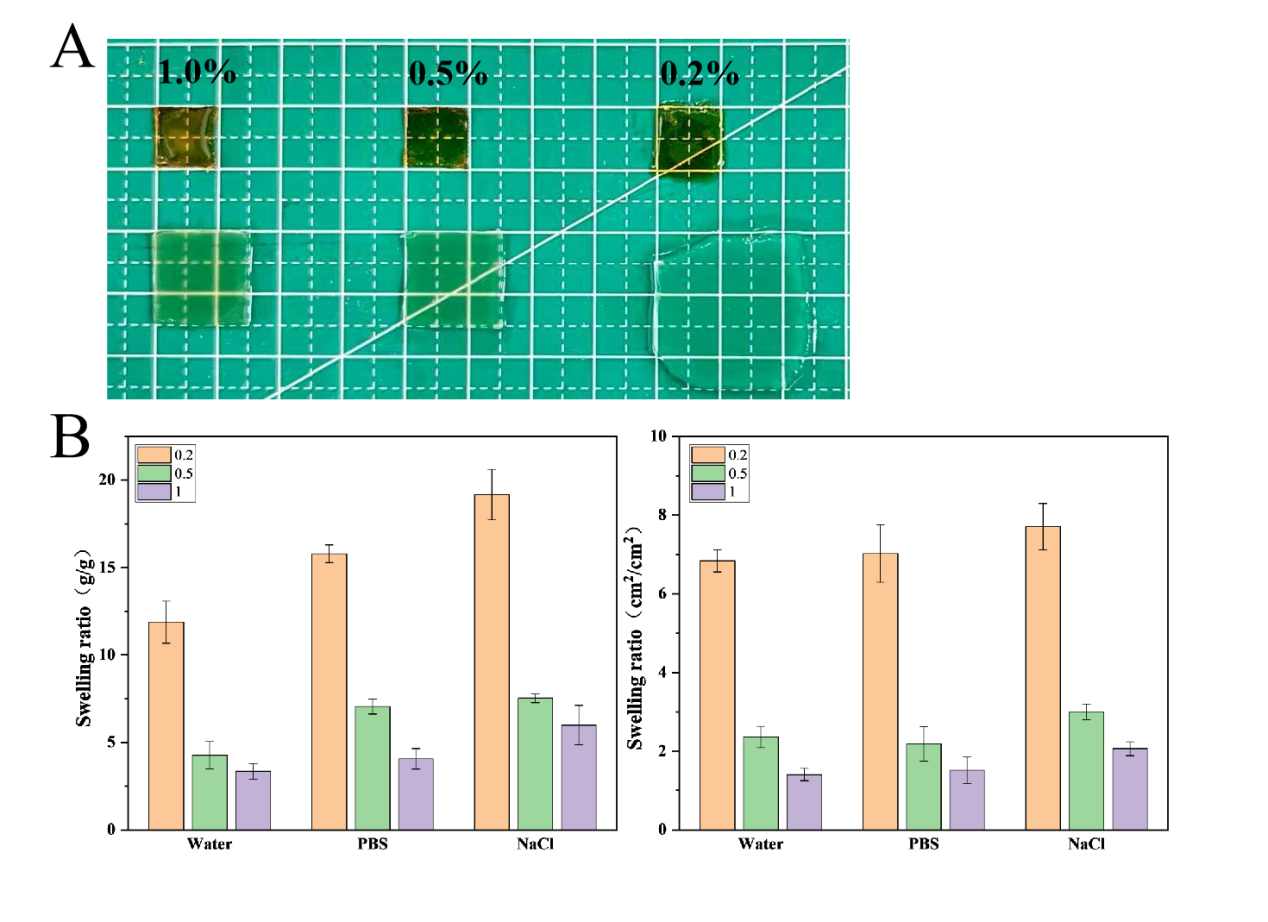


**Figure S5.** (A) Digital photos of the PDA-silk-PAM hydrogels with different crosslinking degrees (Bis/AM) before and after being fully immersed in water solution. (B) Swelling ratio（Left, g/g ; Right, cm^2^/cm^2^）of the PDA-silk-PAM hydrogels with the crosslinking degrees (Bis/AM) of 0.2%, 0.5% and 1.0% fully immersed in water, PBS and NaCl solution. Data are presented as the mean ± SEM, n=3.


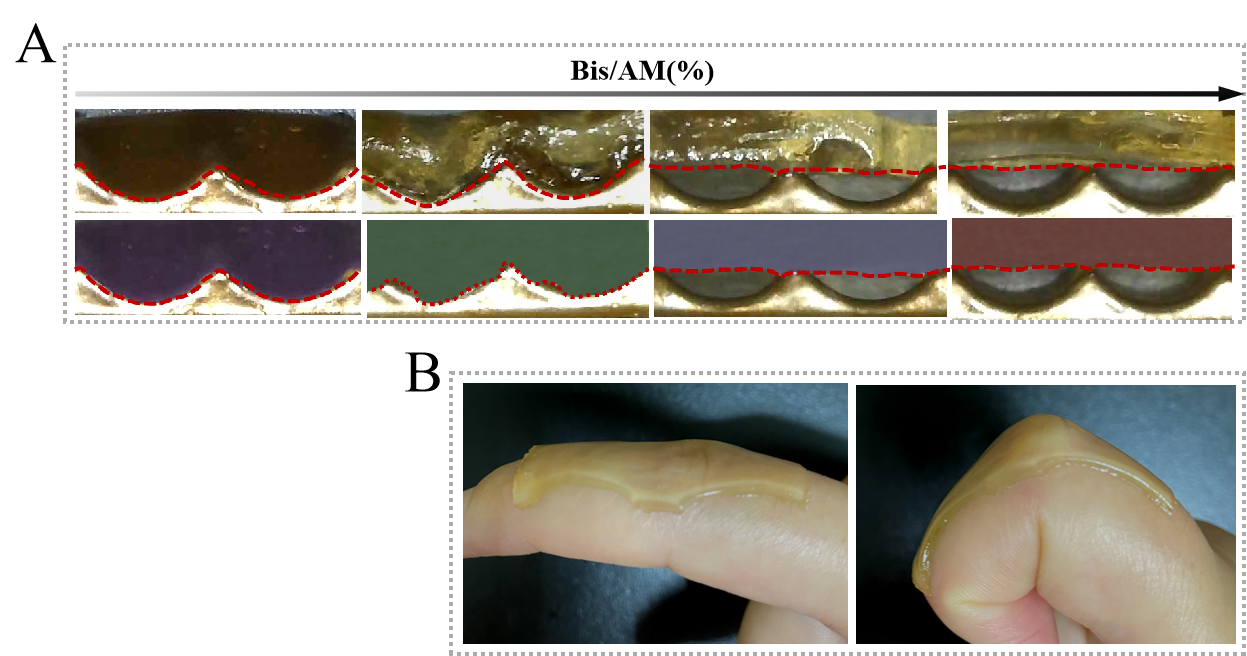


**Figure S6.** (A) Digital photos and schematic photos of PDA-silk-PAM hydrogel films with the crosslinking degrees (Bis/AM) of 0.1%, 0.2%, 0.5% and 1.0% placed on a U-groove line patterns for 12 h in the ambient environment. (B) Digital photos of PDA-silk-PAM hydrogels covered the phalangeal joint.


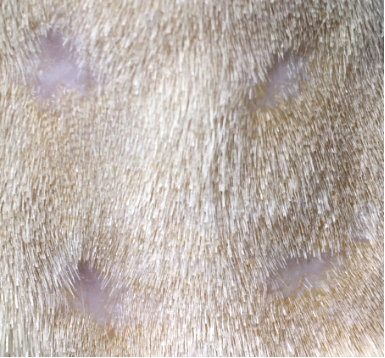


**Figure S****7.** Digital photos of wound healing at 16th day.


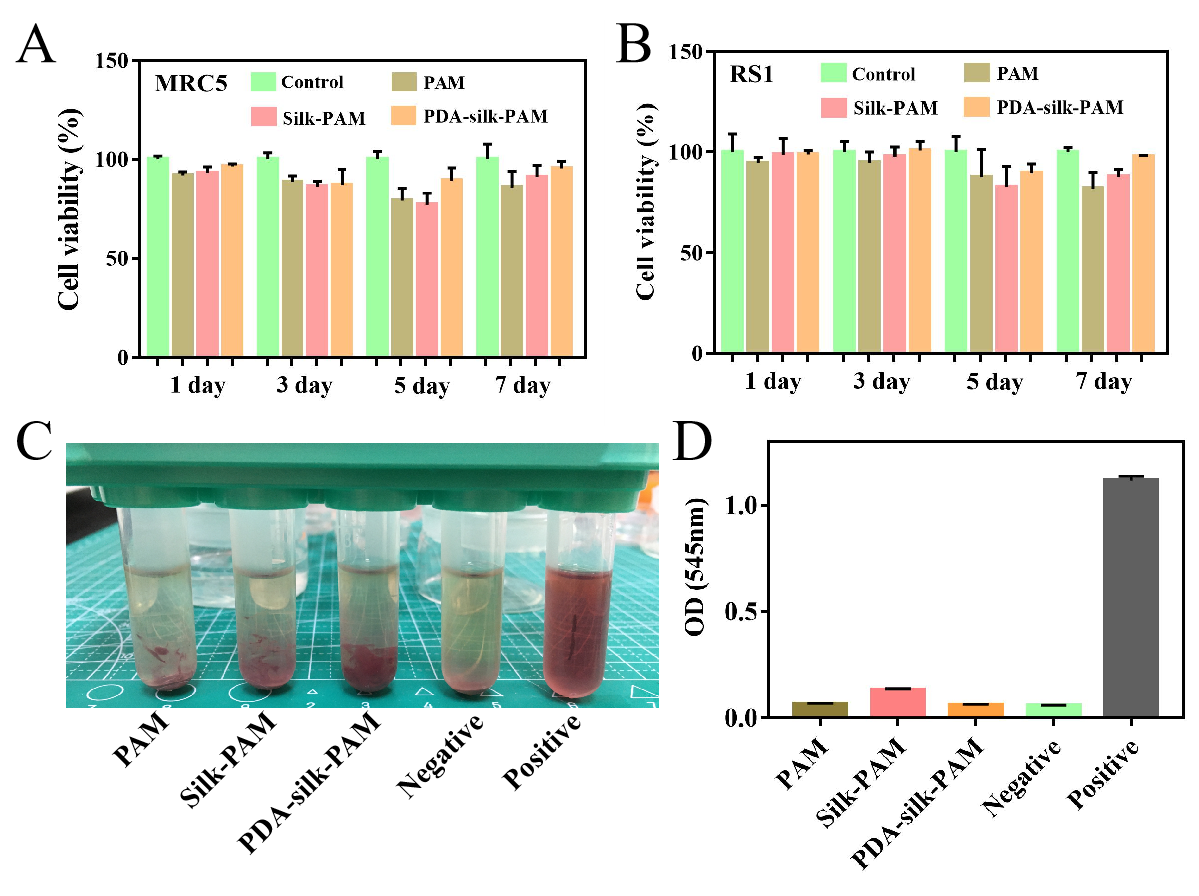


**Figure S8.** Biocompatibility of PDA-silk-PAM hydrogels both in vitro and in vivo. (A) Bar graphs showing viability of MRC5 cells incubated with control, PAM hydrogels, Silk-PAM hydrogels, PDA-silk-PAM hydrogels for 1 day, 3 day, 5 day and 7 day. (B) Bar graphs showing viability of RS1 cells incubated with control, PAM hydrogels, Silk-PAM hydrogels, PDA-silk-PAM hydrogels for 1 day, 3 day, 5 day and 7 day. (C) Hemolytic reaction of red blood cells of PAM hydrogels, Silk-PAM hydrogels, PDA-silk-PAM hydrogels, PBS (negtive), and 0.2% Triton X-100 (positive). (D) Bar graphs of blood compatibility of PDA-silk-PAM hydrogels.


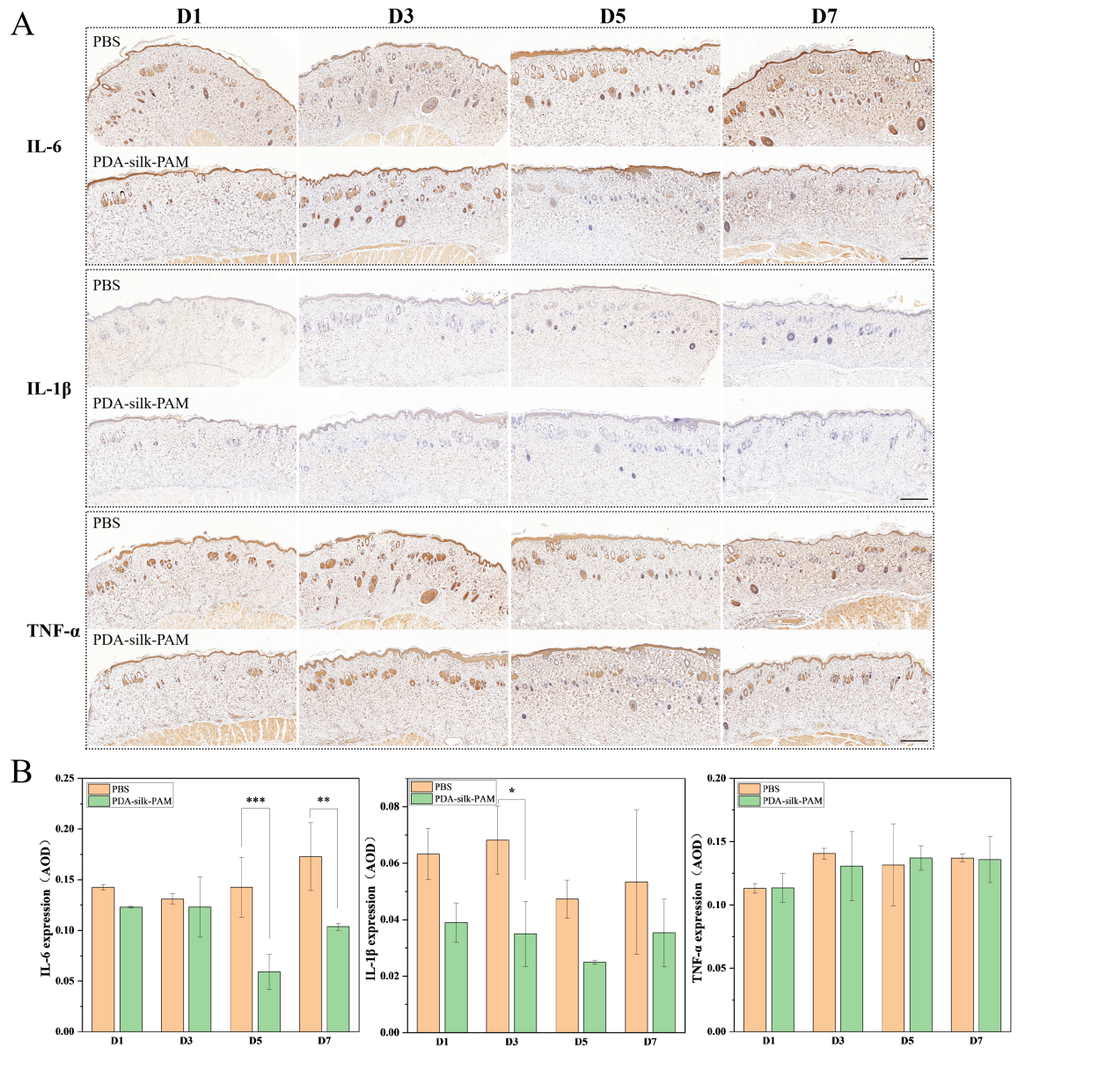


**Figure S9.** (A) Immunohistochemistry staining of IL-6, IL-1β and TNF-α of the skin wounds on the back of rats at the 1th , 3th , 5th and 7th day. Original figures, scale bar: 500 μm. (B) Average Optical Density (AOD) analysis of IL-6, IL-1β and TNF-α of the skin wounds on the back of rats at the 1th , 3th , 5th and 7th day. (Statistical significance was tested using a two-way ANOVA with Bonferroni’s multiple comparisons test; n = 3 for each group; The data represented means ± SEM; *p < 0.05; **p < 0.01; ***p < 0.001.)
